# Cognitive diagnostic assessment of EFL learners’ listening barriers through incorrect responses

**DOI:** 10.3389/fpsyg.2023.1126106

**Published:** 2023-08-17

**Authors:** Yaru Meng, Ya Wang, Ningning Zhao

**Affiliations:** ^1^School of Foreign Studies, Xi’an Jiaotong University, Xi’an, Shaanxi, China; ^2^Xi’an Chanba No.2 Middle School, Xi’an, Shaanxi, China

**Keywords:** cognitive diagnostic assessment, EFL listening barriers, incorrect responses, cognitive diagnostic model, Bug-GDINA

## Abstract

English as Foreign Language (EFL) learners’ cognitive processes have been a research focus in listening assessment. Most studies use correct responses as data, but undervalue the rich information of the incorrect answers or options (in the case of multiple choice questions, MCQ). However, the MCQ distractors are often intentionally designed to reveal learners’ problems or barriers. In order to diagnose the EFL learners’ listening barriers through incorrect responses, Cognitive Diagnostic Models (CDMs) for bugs were adopted, hence the name Bug-CDMs. First, five EFL listening barrier attributes were identified and two Bug Q-matrices were developed to comparatively analyze the learner’s responses with different Bug-CDMs. The results revealed that Bug-GDINA was the optimal model, and the most prevalent barriers were semantic understanding and vocabulary recognition. These barriers confirmed both compensatory and non-compensatory relationships in causing listening comprehension failures. The study proved the feasibility of Bug-GDINA in diagnosing listening barriers from the incorrect responses. Limitations and suggestions for further research were also proposed.

## 1. Introduction

Though listening is a major language skill (Vandergrift and Goh, 2012; Rost, 2016), it is still the thinly profiled, least understood and particularly under-researched skill (Vandergrift and Goh, 2012; Wolf et al., 2019; He et al., 2022), and the learners’ underlying cognitive process is even less addressed (Buck, 2001; Harding et al., 2015). The same is true of Chinese college EFL learners (Xu and Nie, 2016), as a result, understanding their cognitive processes has been a focus in EFL listening research.

Cognitive Diagnostic Assessment (CDA), as a new generation of measurement, has demonstrated its advantages in combining cognitive process and psychometrics (Leighton and Gierl, 2007). It can not only reveal learners’ cognitive processes and mastery or non-mastery of subskills intended by test items (Sawaki et al., 2009), but CDA also helps provide tailored feedback for subsequent remediation and guidance (Yi, 2017; He et al., 2022). This can be achieved by adopting appropriate cognitive diagnostic models (CDMs), which are latent class models used for classifying students based on their skill profiles. Current CDA studies in L2 listening focus on identifying whether a particular learner has mastered some specific language subskills (known as attributes) such as “listening for details” or “listening for main ideas” based on the correct responses or options (in the case of multiple choice questions, MCQ hereafter) (e.g., Min and Xiong, 2019; He et al., 2022), but they undervalue the rich information of students’ incorrect options or distractors which are often intentionally designed and likely to be chosen to reveal specific problems in knowledge or skills (Ozaki et al., 2019), and can also provide useful diagnostic feedback (Stout et al., 2022). Therefore, both incorrect and correct listening responses (or MCQ distractors) deserve equal attention and treatment.

Goh (2000) used a general term “listening problem” to refer to the real-time processing difficulties directly related to cognitive procedures at various stages of comprehension. From cognitive diagnostic perspective in psychometrics, “Bug” is used to refer to misconceptions, lack of skills (Kuo et al., 2016), or the thinking processes interfering with learning (Bradshaw and Templin, 2014). The current study adopts MCQ incorrect responses as data and bug-CDMs as analytical framework to identify the root causes of failures. The distractors often reflect both common problems and misconceptions (Haladyna and Rodriguez, 2013; Jones, 2020), therefore, “listening barriers” (Dunkel, 1991) may be better for the internal and external characteristics to hamper L2 comprehension, though sometimes “problems” will also be used interchangeably.

Up to now, very few attempts were made employing CDA to analyze incorrect responses in L2. One possible challenge might be the lack of appropriate CDMs to accurately identify students’ barriers at sufficiently fine-grained levels (Lee, 2015). Fortunately, Bug-CDMs like Bug-DINO (the bug deterministic inputs, noisy “or” gate mode) (Kuo et al., 2016) were developed, which inspires us to make attempts to analyze the incorrect listening responses. The current study is an early attempt to cognitively diagnose the root causes using Bug-CDMs. The findings would contribute to remedial learning and teaching, test development, and particularly to the design of high-quality MCQ distractors. In the sense, the study is significant methodologically, pedagogically and psychometrically.

## 2. Literature review

### 2.1. EFL listening barriers and the cognitive processes

Listening comprehension is arguably the most complex and multi-dimensional cognitive process (Buck, 2001; Vandergrift and Goh, 2012; Field, 2013), involving both linguistic and non-linguistic competence (Bachman and Palmer, 1996; Buck, 2001). Vandergrift (2007) pointed out that tracking L2 listening barriers is as important as required skills. They can help infer learners’ problematic cognitive processes as well as their interactions. The most systematic study of ESL listening processing problems is by Goh (2000) on Chinese learners in Singapore based on Anderson (1995) three-phase model of perception, parsing and utilization. Perception is the physiological process of receiving auditory signals for processing; parsing is the mapping of the perceived input onto the information from long-term memory, corresponding to bottom-up processing; utilization is drawing on listeners’ world knowledge to fill in gaps in their mental representation of the message, analogous to top-down comprehension. Goh’s 10 problems are derived from the learners’ self-reports, weekly diaries and interviews. The 5 most common problems she identifies are: (1) quickly forgetting what is heard (parsing); (2) not recognizing words or unfamiliar alternative pronunciations of words they know (perception); (3) understanding words but not their intended message (utilization); (4) neglecting the next part when thinking about meaning (perception); and (5) inability to form a mental representation (parsing). Since four of them are in the perception and parsing stage, it may indicate that the fluent listening comprehension is often obstructed by limited lexical and syntactic knowledge (Juan and Abidin, 2013; Vafaee and Suzuki, 2020; Yeldham, 2022). This is further confirmed by Cao et al. (2016) two new types of problems as “Confused about unexpected word appearance” and “Unsure about the meaning of words.” Goh (2000) study made an enormous contribution by establishing the correlation between the three cognitive stages and the learners’ reported difficulties, moving listening barrier research forward. However, there are different versions of this correlation, which has since become an issue (Zhang et al., 2010; Feng and Xu, 2022), hence needing more fine-grained models for this purpose. Since then, different listening comprehension models have been adopted to facilitate a better understanding of the listening process, including listening barriers. These models include Vandergrift and Goh (2012) 3 process model, Field (2013) “two level” model and Rost (2016) four category model. Rukthong and Brunfaut (2019) integrate the above models into 5 sub-skills of acoustic-phonetic decoding, word recognition, parsing, semantic processing, and pragmatic processing, with the former three stages as lower-level processes and the latter two as higher-level processes. This integrated model incorporates top-down and bottom-up, and is also more able to demonstrate the complex and interactive nature of both high-level and low-level cognitive processes involved in L2 listening. Therefore, it may shed light on learners’ barrier identification.

### 2.2. Methods in L2 listening barriers research

To investigate learners’ listening comprehension problems, various procedures and empirical methods have been used, including listening diaries and interviews (early attempts like Goh, 2000), think-aloud protocols (Hwang, 2004), and questionnaires (Zhang and Zhang, 2011; Noroozi et al., 2014). For example, Hwang (2004) studied listening problems with high school students by asking them to verbally describe what they heard, and then reflected on and wrote down any barriers they encountered in listening. These retrospection techniques provide opportunities for listeners to recall the listening experience and offer insights into their listening processes at a later moment in time, though the reliability of their results is limited by the reliability of the measurements or, in the case of the recall protocols, inter-rater reliability. Listening barrier analysis based on test scores tells us something about the outcome based on correct answer, i.e., the level of listening success, and may verify comprehension, but it reveals very little about how students arrived at comprehension or, more importantly, how comprehension failed. In view of this, many scholars have adopted the analysis of wrong responses to diagnose learners’ lack of skills and help overcome their difficulties. This resembles error analysis, while in assessing L2 listening problems, error analysis is most often focused on dictation tasks (Cho, 2021). Kao and Kuo (2021) used 12 MCQ items from TOEIC Bridge test and asked learners’ to self-report on their incorrect listening items for diagnosing the real sources of their problems in EFL contexts. They also used Goh (2000) top five problems for mediation moves, but their insufficient psychometric-based validity evidence may have caused inaccurate diagnoses. On the other hand, this use of wrong options in MCQ items for future mediation inspired further attempts.

The diagnosing power of MCQs, and especially the messages in their options, was pointed out by Briggs et al. (2006). He demonstrated that multiple-choice items based on construct can provide diagnostic information. Andrade and Heritage (2017) made one more step forward by saying that “When each of the possible answer choices in an item is linked to student understanding, an item-level analysis of student responses can reveal what individual students know” (P.46). It’s true that the wrong choices are not simply wrong. Each is wrong in a way that reflects a common gap or misconception. There is some evidence that diagnostic, multiple-choice items are actually better than open-ended items at eliciting students’ true understanding, perhaps because the items probe students’ thinking by offering plausible incorrect answers (Steedle and Shavelson, 2009). The incorrect answers that demonstrate incomplete understanding, errors in reasoning, or misconceptions are useful to teachers, who can use them to identify next steps in instruction (Andrade and Heritage, 2017). But precisely how the incorrect options in listening tests reflect the learners’ cognitive processes and barriers is an area seldom ventured into. Cognitive Diagnostic Assessment, as one recent psychometric development, prides itself on combining qualitative and quantitative evidence for fine-grained validation and feedback, thus giving it potential for tapping into the diagnostic information from incorrect options.

### 2.3. Cognitive diagnostic assessment for listening barriers

Compared with the traditional tests that simply rank-order examinees on a one-dimensional continuum, Cognitive Diagnostic Assessment (CDA) can comprehensively investigate the multidimensional cognitive processes, thus inferring the non-observable knowledge state of an individual based on the observable response data (Leighton and Gierl, 2007; Rupp et al., 2010). It can explore learners’ differences in internal cognitive processes or knowledge structures so as to offer pedagogically useful information for subsequent learning and teaching remedies (Jang et al., 2015; Chen and Chen, 2021). Under the framework of CDA, three major iterative procedures should be undertaken in order to obtain diagnostic results about learners’ specific abilities, namely, cognitive attribute identification, Q-matrix construction, and data analysis. Cognitive attributes refer to the cognitive skills, strategies, and knowledge that learners might need to correctly complete a given task. They are often associated with test items and their relationships are represented in a two-dimensional incidence called a Q-matrix, which is expressed with a “0” or a “1,” indicating an item not requiring or requiring an attribute (Rupp et al., 2010). In conjunction with learners’ item response data, the Q-matrix is then inputted into certain psychometric models called cognitive diagnostic models (CDMs) for data analysis during which model-data fit statistics are evaluated. If a mismatch is identified, one should revise the primary attributes and the Q-matrix until an appropriate fit is achieved. With all those procedures completed, the diagnostic results showing students’ mastery profiles can be obtained both at group and individual levels. Due to CDA’ s great potential for discovering learners’ underlying performance, it has been widely used in the field of language assessment.

Most CDA-based listening studies explore what attributes can best represent L2 listening ability (Sawaki et al., 2009; Meng, 2013) and the underlying inter-attribute relationships (Yi, 2017; Min and Xiong, 2019; Dong et al., 2021). Therefore, the target attributes of most studies are listening sub-skills. No wonder Harding et al. (2015) worried that the regular CDA could not help find the root causes of students’ barriers. To address this, careful attention should be given to incorrect options, to exploit diagnostic information and pinpoint students’ cognitive problems. Kuo et al. (2016, 2018) employed Bug-CDMs to diagnose students’ cognitive problems based on their incorrect responses in mathematical multiple-choice items, proving the feasibility of using Bug-CDMs for problematic cognitive diagnosis, and thus inspiring us to consider their application in the field of language testing. In other words, Bug-CDMs may have the potential to assist in diagnosing listening comprehension problems.

Similar to the basic assumptions of the normal GDINA models, all bug-related models are realized through the modification of the latent variables into “bugs,” and can then be used to analyze the bug data. But the non-saturated models like Bug-DINA or Bug-DINO include only specific parameters, hence with limited generality. For example, with Bug-DINO model, a learner cannot get a correct answer if he possesses one or more barriers the item measures (Kuo et al., 2016, 2018); with the Bug-DINA model, if and only if a learner has all the barriers the item measures, will he be more likely to get the answer wrong. In contrast, Bug-GDINA, parallel to the GDINA model which is commonly used in CDM studies and accommodates all the possible major and interactive effects between the attributes (Rupp et al., 2010), has the same advantages and can also realize greater generality. But Bug-GDINA is also often penalized for violating the principle of parsimony for model application with its inclusiveness.

### 2.4. Research questions

Bug-CDMs’ development and application are in their infancy even in psychometrics, and their potential in solving EFL learning problems has not yet been explored. So the current study attempts to tap the feasibility of Bug-CDMs in diagnosing listening processing problems through the incorrect options. To accomplish this purpose, the following two research questions are addressed:

RQ1: To what extent can cognitive diagnostic assessment identify EFL listening barriers?

RQ2: To what extent can diagnostic results help reveal the relationships between listening barrier attributes?

## 3. Methods

### 3.1. Participants

A total of 1,121 EFL college students (approximately 17–19 years old) with intermediate language proficiency (National Matriculation English Test, roughly equivalent to CEFR B1 level) participated in the listening test. They were all freshmen with an average of over 6 years of prior EFL learning experience, and were recruited from six universities at different levels (2 top-tier, 3 s-tier and 1 third-tier) in western China. Six and twelve test takers, respectively, volunteered to participate in the first and second verbal protocol sessions, with the former targeted at modifying the identified attributes, and the latter aiming to validate the Q-matrix. In addition, eight content experts were invited to code the incorrect options as barrier attributes. These experts not only had considerable experience in language test development and teaching, but were also familiar with cognitive diagnostic approaches.

### 3.2. Instruments

The diagnostic listening tests were selected from the item pool of PELDiaG system (Personalized English Learning Diagnosis and Guidance system) designed for diagnostic purposes (Meng, 2013; Dong et al., 2021; Meng and Fu, 2023) and the CET 4 test. The Cronbach’s alpha of the test is 0.724, and the KMO is 0.848 (*p* < 0.001), indicating good reliability. The item selection process involved two steps: first, IRT (Item response theory) analysis was conducted to obtain the item parameters. Items with good discrimination (a > 0.3) and difficulty (3.0 ≥ b ≥ −3.0) were chosen. Afterwards, the quality of incorrect options was analyzed through the response frequency, which means that the distractors with low-frequency (<5%) were screened out (Haladyna and Downing, 1993). In the end, a total of 19 multiple-choice items were selected. There were three sections with 11 short dialogue items, 1 long conversation, and 2 passages. The topics covered familiar ones like shopping, education, public transportation and technology. The tests were administered in paper-and-pencil format at regular class times, and the responses were scored dichotomously.

Five Bug-CDM models were compared for the optimal model-data fit, including Bug-DINO, Bug-DINA, Bug-GDINA, Bug-RRUM, and Bug-ACDM. The comparison was done using the “GDINA” package (version 2.9.3) (Ma and de la Torre, 2020) embedded in R studio, which provides a platform for a series of cognitively diagnostic analyses for dichotomous and polytomous responses.

### 3.3. Procedures

The procedures in this study bore a strong resemblance to those in diagnosing listening skills which included five major stages: identifying the attributes, constructing the Q-matrix, validating the Q-matrix, selecting optimal CDM and generating feedback.

#### 3.3.1. Identifying the barrier attributes

First, the researchers proposed a preliminary list of common EFL listening barriers based on listening cognitive processing models and literature review. Then, 12 students were recruited to participate in verbal reports for their encountered barriers in finishing the target listening items, according to which the authors’ barrier attribute names and definitions were derived. For example, the fifth attribute B5 “barrier in making pragmatic inferences” was modified because some students reported the overuse of prior knowledge. The following excerpt from Participant 1, who answered the item (Item 16) in a passage incorrectly, might help to illustrate.

Item 16: What can be inferred from the passage?

- **Helping others brings positive emotions. (Correct Answer)**
- Volunteering benefits the receivers more.
- Everyone needs help and friends.
- Helping others means power.

*Transcript: … Volunteer is to help, and when we help others, we satisfy our needs to have a degree of control over our world. When people see smiles and satisfaction in person being helped, they can feel happier in their hearts…*

Participant 1: *“In fact, I haven’t grasped all information the speaker mentioned here, but I believe if the volunteer service is provided, many people (receivers) can get benefits from it. So I decide to choose option B.”*

In this case, the barrier impeding participant 1 was not the lack of sufficient prior knowledge, as this barrier is often interpreted. Rather, he over-used his prior knowledge about the volunteer service, resulting in an over-generalization and his neglect of key information. In other words, since he had not fully understood the original information, he was prone to overusing background knowledge. This attribute was accordingly redefined as “barrier due to the lack of or over-generalization of prior knowledge.” Students’ verbal data also revealed that sometimes the incorrect answer may not result from a single barrier, but the interactive effect of two or more barriers. For example, when participant 1 was trying to solve item 3, he not only overgeneralized prior knowledge (B5), but also missed the explicitly expressed information (B4). In short, with verbal data analysis, the barrier attributes were modified and validated. See Table 1 for the final version.

**Table 1:** Modified barriers.

Cognitive models	Related research	Verbal reports excerpts	Barrier attributes	Definition of barrier attributes
Acoustic- phonetic decoding	Goh (2000), Graham (2006), Namaziandost et al. (2019), and Nushi and Orouji (2020)	“I clearly heard ‘camp.’” (But the mentioned word is “cab”)	B1: Identifying speech	Unable to identify the auditory, phonetic, and phonological features.
Word recognition	Goh (2000), Juan and Abidin (2013), Cao et al. (2016), Namaziandost et al. (2019), Vafaee and Suzuki (2020), and Alharbi and Al-Ahdal (2022)	“… I did not catch most of the words in the text….”	B2: Recognizing vocabulary	Unable to identify words or phrases in a speech stream or activate the relevant word knowledge.
Parsing	Goh (2000), Cao et al. (2016), and Vafaee and Suzuki (2020)	“…I got all the words in the sentence, but I still couldn’t understand the meaning of the sentence.”	B3: Understanding syntactic or semantic structures	Unable to understand the syntactic or semantic structures of the language to generate the local representations of text (clause level).
Semantic processing	Goh (2000), Graham (2006), and Cao et al. (2016)	“… I just follow the audio to understand the specific contents, but ignore to connect with the information speakers have mentioned before, so it's hard to get the general idea of the paragraph.”	B4: Understanding semantic meanings	Unable to identify or synthesize explicit information at multiple locations to generate the coherent representations of text (discourse level).
Pragmatic processing	Goh (2000), Graham (2006), Juan and Abidin (2013), Cao et al. (2016), and Alharbi and Al-Ahdal (2022)	“(I believe) If the volunteer service is provided, many people will get benefits from it.” This student overextended his prior knowledge about volunteering instead of referring to text's original contents.	B5: Making pragmatic inferences	Unable to infer implicit contents due to the lack of or over-generalization of prior knowledge, and misunderstanding texts’ linguistic information and communicative contexts.

#### 3.3.2. Constructing the preliminary Q-matrix

The development of the Q-matrix was informed by two sources of information: the final version of barrier attributes and expert judgment. First, eight experts were invited to participate a Q-matrix coding training workshop on how to analyze the MCQ distractors for barriers. Then they individually decided whether a certain barrier (or barriers) was involved in each incorrect option for an item, based on the examination of the above barrier definitions. According to the attribute coding results, if more than half of experts reached an agreement on a certain barrier, then it was identified as a listening barrier for the item. Otherwise, the barrier was rejected. Hence the preliminary Q-matrix (Q1) was developed (see items for modification on Table 2).

**Table 2:** The final Q-matrix.

Item	Ob1	Ob2	Ob3	Ob4	Ob5	Items	Ob1	Ob2	Ob3	Ob4	Ob5
Item1	1	0	0	0	0	Item11	**1**	0	0	1	0
Item2	1	1	0	0	0	Item12	0	0	0	1	1
Item3	0	1	0	1	0	Item13	0	1	0	**1**	1
Item4	1	1	0	1	0	Item14	0	0	0	0	1
Item5	0	0	1	0	0	Item15	0	0	**1**	1	1
Item6	0	0	1	1	1	Item16	0	0	0	1	1
Item7	0	0	1	1	0	Item17	0	0	1	1	1
Item8	0	0	1	1	0	Item18	0	0	0	1	1
Item9	0	0	0	1	0	Item19	0	0	0	1	1
Item10	0	0	0	0	1						

1 indicates the corresponding barrier is measured by a particular item. 0 indicates otherwise. Bold font numbers indicate revisions from the preliminary Q-matrix.

#### 3.3.3. Validating the Q-matrix

In order to modify the item-barrier mapping, two steps were taken: verbal report 2 and the data-driven method. In terms of the former, an example is provided here to illustrate the process.

Item 11: What can be inferred about Phillip?

- He’ll go to the party with the woman.
- He will not meet the man at the party.
- **He has changed his plans. (Correct Answer)**
- He has to work late.

*Transcript:*

*(Woman): I talked to Phillip today, and he said he’ll be come to the party.*

*(Man): So he CAN come after all.*

Originally, the expert-coded barrier was “understanding semantic meanings” (B4), which meant the listener probably chose the wrong answer when he could not understand or synthesize the speakers’ intention. Nevertheless, further evidence was collected through interviewing participants. For example, participant 2 stated:

*“… the woman said Phillip will come to the party, and then the man probably agree with her because he said Philip can come. But I’m not very sure about this … it’s a little bit like he can’t come. In fact, I didn’t catch clearly what the man said is **can or can’t**.”*

According to this participant, one of the barriers he encountered was that he could not distinguish the phonetic features between “can” and “cannot,” and therefore, we added the attribute B1 in our preliminary Q-matrix 1.

To empirically revise and validate the Q-matrix, the data-driven method was employed using the GDINA package. Based on the initial analysis, suggestions for revision of the Q-matrix were put forward. For example, it was suggested that B4 “barrier in understanding semantic meanings” be deleted for item 11. Similarly, for items 13 and 15, B3 “barrier in understanding syntactic or semantic structures” was recommended. But considering that psychometric analysis should not be the only sources for Q-matrix revision (Aryadoust and Luo, 2022), the study also included expert judgment. The experts were asked to analyze the items and suggested barriers, and then to reach a consensus after discussion. Final revisions to some of the items were then completed (see Table 2 for Q-matrix 2). Again, taking item 2 as an illustration, when it was not successfully answered, it was more likely that students were unable to identify the phonetic features, and at the same time had difficulties in activating word knowledge.

#### 3.3.4. Selecting the optimal CDM

As mentioned above, five Bug-CDMs were employed and compared in order to choose the optimal model. Both relative and absolute fit statistics were obtained (see Table 3). In terms of the former at the test level, the maximum z-scores (denoted as zr) of the residual between the observed and predicted Fisher-transformed correlation of item pairs was produced; in terms of the latter, the residual between the observed and predicted log-odds ratio (LOR) of item pairs (denoted as zl) were produced. It can be seen that the Bug-GDINA has the better model-data fit (Max zr =3.3777, *p* = 0.125 > 0.05; Max zl = 3.4044, *p* = 0.113 > 0.05), followed by the Bug-ACDM (Max zr =3.5097, *p* = 0.08 > 0.05; Max zl = 3.5591, *p* = 0.06 > 0.05). However, BIC for Bug-GDINA was not the lowest, since it generally imposes a penalty on highly parameterized models (Murphy, 2012). But overall, based on the values of absolute fit and relative fit, as well as the saturated model’s capacity in identifying complex relationships of listening comprehension, Bug-GDINA proved to be the optimal one for further analysis.

**Table 3:** Model fit comparison of Bug-CDMs using the final Q-matrix.

Bug-CDMs	Npars	Relative fit			Absolute fit	
		−2LL	AIC	BIC	Max zr	Max zl
Bug-DINO	69	26126.38	26264.37	26610.83	4.1628	4.2254
Bug-DINA	69	26126.5	26264.49	26610.95	4.1191	4.2199
Bug-GDINA	133	**25822.3**	**26088.3**	26756.1	**3.3777**	**3.4044**
Bug-RRUM	91	25941.44	26123.44	**26580.36**	4.0201	3.9800
Bug-ACDM	91	25945.94	26127.95	26584.86	3.5097	3.5591

Critical z-score = 3.649, 4.044 for α = 0.05, 0.01, respectively (with the Bonferroni Correction) (Chen et al., 2013). Bold font indicates optimal fit values.

## 4. Results

RQ1: To what extent can cognitive diagnostic assessment identify EFL listening barriers?

**Figure 1:**
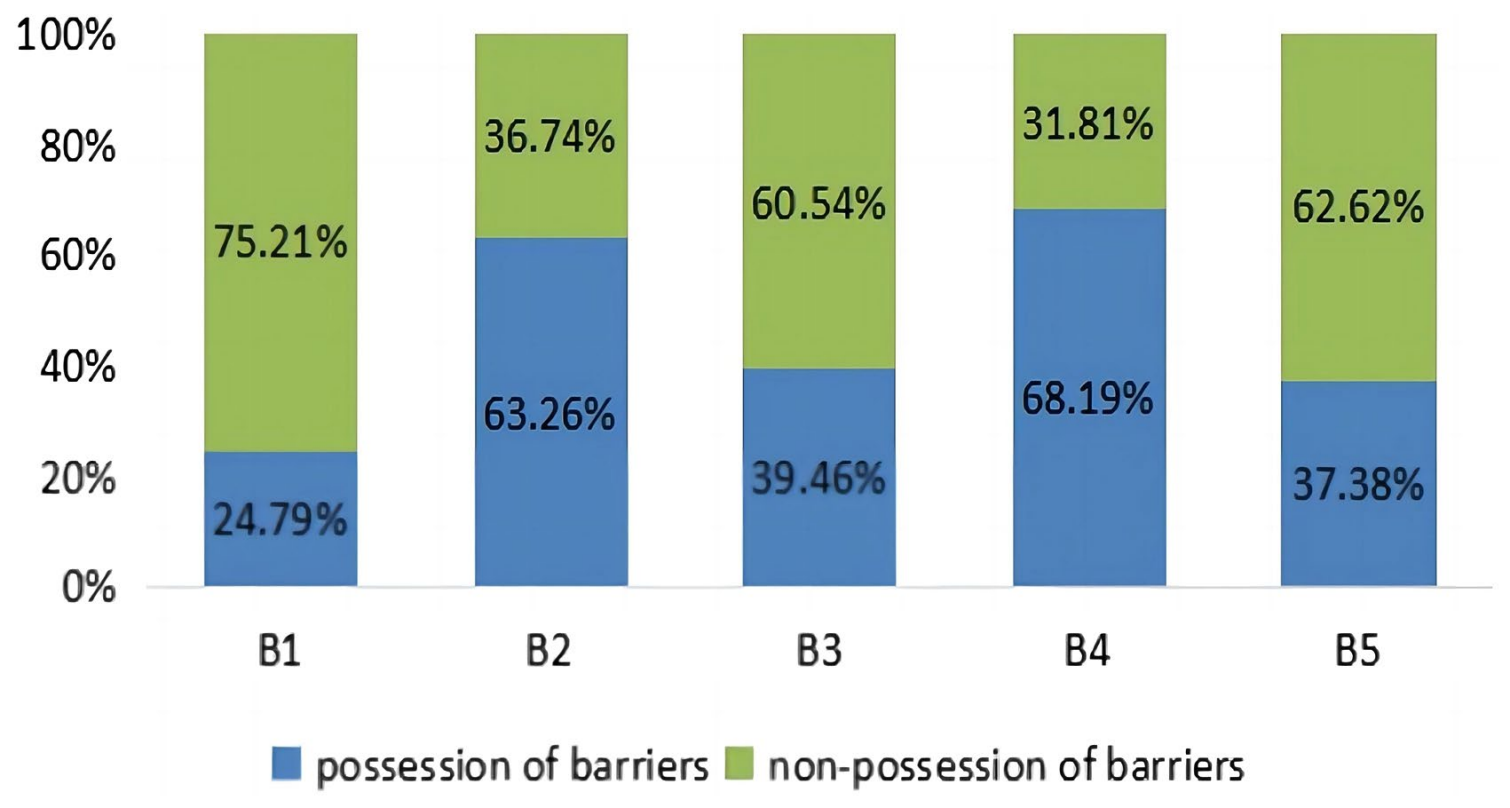
Attribute prevalence.

At group-level, we can see in Figure 1 the overall barrier profile for students’ listening comprehension through “attribute prevalence,” which shows learners’ mastery probability of each barrier attribute, ranging from 24.79 to 68.19%. As Figure 1 makes clear, “understanding semantic meanings” (B4) is the most prominent barrier impeding learners’ listening comprehension, while “identifying speech” (B1) is the least challenging factor. The second most serious problem (63.26%) is a difficulty in “recognizing vocabulary” (B2). The probabilities of encountering barriers in “understanding syntactic or semantic structures” (B3) and “making pragmatic inferences” (B5) were fairly close together (39.46 and 37.38%, respectively), suggesting their similar hindering effects on learners’ listening comprehension.

At individual-level, all students were classified into different latent classes. With 5 attributes in the current study, a total of 32 theoretically possible latent classes were identified. The top eight most dominant patterns are presented in Table 4. The five numbers in each latent class correspond to 5 barrier attributes, again with “1” denoting the presence of a certain barrier and “0” otherwise. As is shown, two flat profiles “11111” and “00000” enjoy the top percentages, indicating the possession of all the attributes is the most popular profile, and the second one is with none of these attributes. The remaining six jagged profiles demonstrate learners’ strengths and weaknesses (He et al., 2021). When only one barrier is possessed, the latent class profiles were more likely to be 00010 (15.24%) and 01000 (9.04%), as well as a relatively high percentage of 01010 (12.22%), all indicating the prevalence of B2 and B4 in the current sample. Latent class profiles “01110” and “11001” mean that those learners have three barriers, with the class probabilities of 5.13 and 1.67%, respectively. In the same vein, the profile “01111” suggests that learners (11.94%) possess four barriers with the exception of B1, meaning that it is probably the least prevalent barrier.

**Table 4:** Eight dominant latent classes and posterior probabilities.

Latent class	Posterior probability	Latent class	Posterior probability
11111	18.43%	01111	11.94%
00000	17.10%	01000	9.04%
00010	15.24%	01110	5.13%
01010	12.22%	11001	1.67%

**Figure 2:**
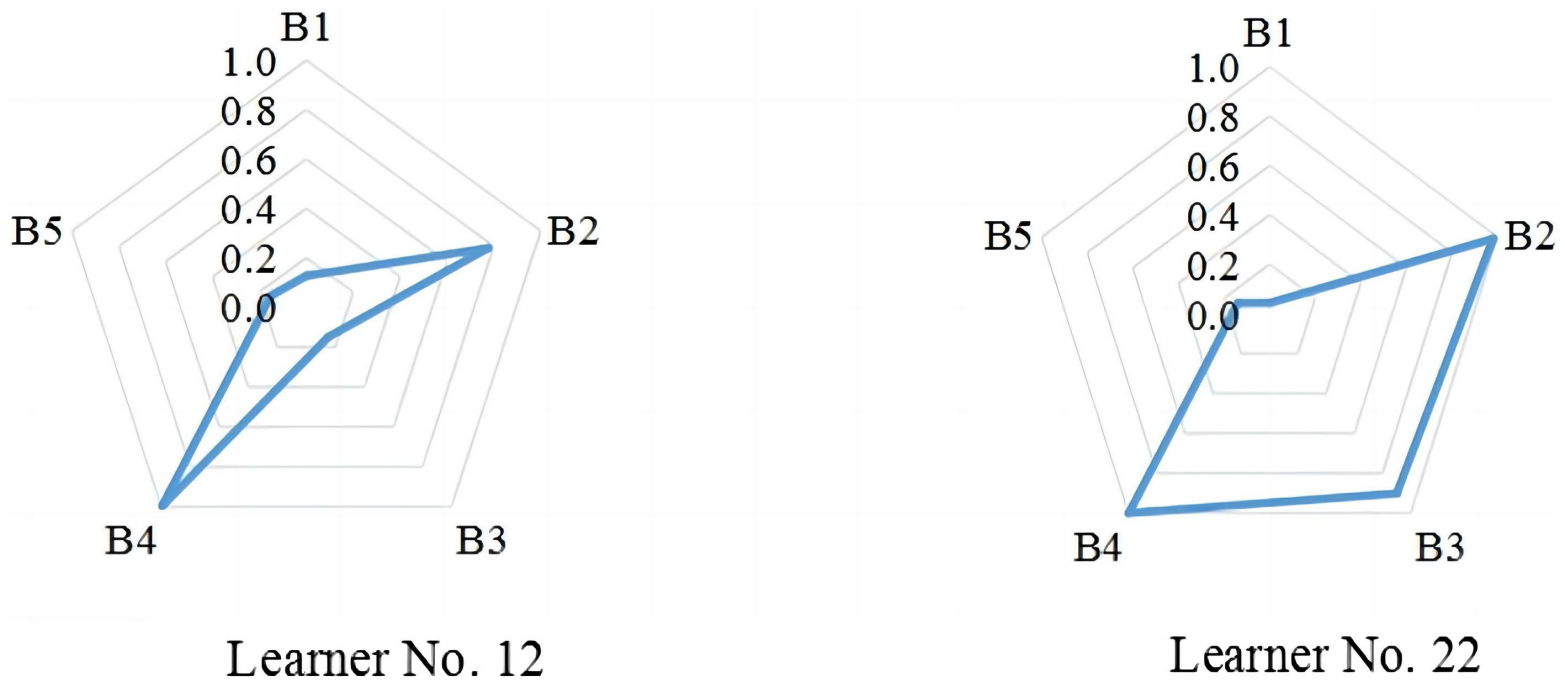
Different person parameter estimation of learners with the same total score.

However, it is worth noting that the 0/1 classification may overgeneralize learners’ knowledge states to some degree (Du and Ma, 2021). Considering this, “person parameter estimation” was used to represent to what extent individual learners possessed a certain barrier attribute, especially with the same total scores. This further reveals the personalized differences, as Learner No. 12 and No. 22 illustrate in Figure 2. They exhibit different personal attribute patterns, though both achieved the same total score of 12. The major barriers for Learner No. 12 were B2 and B4; while for learner No. 22, B2, B3, and B4 were the main issues obstructing listening success.

RQ2: To what extent can diagnostic results help reveal the relationships between listening barrier attributes?

In terms of the inter-relations between the barrier attributes, the tetrachoric correlations between the attributes and the item profiles can be obtained from the Bug-GDINA estimation, as shown in Table 5. The attribute tetrachoric correlation is based on the base-rate probabilities of attribute mastery (Toprak and Cakir, 2021). Generally, values above 0.70 are regarded as strong, 0.50–0.70 as moderate, and below 0.50 as weak (Aryadoust, 2018). In this study, moderate to strong correlations between barrier attributes were identified. As indicated, 5 out of 10 (50%) were strongly correlated (see the bold values), suggesting their strong tetrachoric correlations. For example, B3 exhibited moderate to strong relationships with all the other attributes, probably indicating that B3 alone could not cause listening failure, whereas when combined with other attributes, it may hinder students’ listening cognitive processing (as learner No. 22’s case in Figure 2). However, the correlation coefficient between B2 and B4 was the lowest (0.44), suggesting a certain degree of independence, which is also demonstrated in the case of learner No. 12.

**Table 5:** Tetrachoric correlations between the attributes.

	B1	B2	B3	B4	B5
B1	1				
B2	0.53	1			
B3	**0.76**	**0.83**	1		
B4	**0.75**	0.44	0.62	1	
B5	0.58	**0.79**	**0.92**	0.62	1

The bold values in table show strong tetrachoric correlations.

To illustrate how the interrelationships between attributes can lead to an incorrect response to one particular item, four item profiles extracted from the Bug-GDINA estimation are presented in Table 6. Column 3–6 demonstrate the probability of an incorrect response to each item under different attribute patterns. As is shown, item 3’s failure mainly results from B2 and B4, and the learner’s possession of either of the two attributes or both would indiscriminately result in a high probability of a wrong response. This indicates the non-compensatory relation between B2 and B4. This is also the same for item 8. In contrast, B2 and B5 in item 13 demonstrate a compensatory relationship because the probability of an incorrect response is much lower when either barrier is present, but only when both barriers occur simultaneously do learners commit an error. The same is true for B1 and B4 in item 11.

**Table 6:** The four selected item profiles.

Item	Attribute Pattern	P(00)	P(10)	P(01)	P(11)
3	B2-B4	0.12	0.83	0.89	0.94
8	B3-B4	0.29	0.65	0.74	0.88
13	B3-B5	0.22	0.52	0.45	0.78
11	B1-B4	0.18	0.34	0.42	0.76

P (00) represents the non-possession of neither barrier, P (10) represents the possession of the first barrier attribute, P (01) represents the possession of the second barrier attribute, and P (11) represents the possession of both barriers.

Item 8 below is taken as an example to further explain the relationship between B3 and B4. If learners could not understand the syntactic structure of the subjunctive mood (B3) in the woman’s utterance, it would be difficult for them to choose the correct option A. Otherwise, they would choose an incorrect answer, such as option D, based on irrelevant common knowledge (because the bad traffic may also lead to traffic accidents). Only when learners understand and synthesize utterances by both the man and woman, can they generate coherent representations (B4) and have a high probability of choosing the correct answer, indicating their non-compensatory interaction.

Item 8: What happened to the speakers?

**(A) They missed the train because of the bad traffic. (Correct Answer).**

(B) They arrived at the railway station just in time.

(C) They barely caught the bus to the railway station.

(D) They had a traffic accident on the way to the station.

*Transcription:*

(Woman): If the traffic wasn’t so bad, we could have arrived at the railway station in time to catch the train.

(Man): What a shame! We’ll have to wait for the next train.

## 5. Discussion

Unlike previous research on skill-based CDA in the language domain, this study set out to explore the feasibility of employing Bug-CDMs to investigate EFL learners’ cognitive processing barriers in listening. The five major barrier attributes include “identifying speech” (B1), “recognizing vocabulary” (B2), “understanding syntactic or semantic structures” (B3), “understanding semantic meanings” (B4), and “making pragmatic inferences” (B5). Comparison analysis of five available Bug-CDMs revealed that Bug-GDINA was best fitted to the data. That means it carries the same qualities of a saturated model as GDINA, showing itself to be the most suitable model for uncovering the attributes of the underlying barriers affecting listening comprehension and their interactions with each other. Moreover, from the different mastery profiles of two cases (No. 12 and No. 22) with the same score, this study also stresses the advantage of Bug-CDMs in providing fine-grained diagnostic information, facilitating both instructors and learners with group and individualized feedback for the future improvement of their most problematic skills (Jang et al., 2015).

### 5.1. EFL learners’ major listening barriers

By employing Bug-CDMs, the study addresses Chinese EFL learners’ listening barriers. It contributes to a better understanding of different cognitive demands. The results reveal that the barriers in “understanding semantic meanings (B4)” and “recognizing vocabulary (B2)” are found to be the most prevalent causes for comprehension failure, followed by barriers in “understanding syntactic or semantic structures (B3),” “making pragmatic inferences (B5),” and “identifying speech (B1).” In general, they are quite distinct from the traditional listening problems defined in some studies such as “fast speed of delivery and difficulties in concentrating” (Flowerdew and Miller, 2005), “the shortage of background knowledge of English vocabulary” (Juan and Abidin, 2013), and “misunderstandings of speakers’ accents” (Namaziandost et al., 2019). Possible reasons for the differences may be found in the different theoretical rationales and classification criteria. The current study is, first of all, based on the cognitive processing model, which is top-down oriented. At the same time, bottom-up evidence like learners’ verbal reports serves as complementary evidence. In addition, the thus identified barrier attributes are mapped onto the learners’ incorrect options to produce a bi-dimensional Q-matrix with emphasis on the stable cognition level of sustained misunderstanding or misuse. These multiple sources of evidence for Q-matrix construction ensured the reliability and validity of diagnostic inferences (Jang, 2009). By contrast, most listening problem studies are based on questionnaires, verbal reports, diaries or interviews (e.g., Goh, 2000; Graham, 2006; Nushi and Orouji, 2020; Alharbi and Al-Ahdal, 2022), which cannot establish such close relations between wrong answers and listening mechanisms.

Though different in terms, the current results do not radically deviate from the previous findings. As mentioned above, the most frequent barrier is understanding semantic meanings at discourse level (B4), suggesting that students find it difficult to generate a coherent representation of the processed utterance. This, to a large extent, corresponds to the problem “unable to form the mental representation of words heard” by Goh (2000) and Zhai and Liu (2010), since both are about meaning construction barriers. Results from verbal reports in this study further showed that although some learners were able to handle the semantic structures (B3) to generate the local representations at sentence level, it was still very challenging for them to connect the text already processed with the incoming new text (Cao et al., 2016). This finding confirms what Becker (2016) stated: analyzing and connecting different pieces of information often involves extra cognitive load and thus increases the difficulty of listening comprehension. This overloading is also due to the multitasking nature of listening, as Field (2009) as well as Zhai and Aryadoust (2022) pointed out, when listeners are presented with concurrent audio input, item stem and option reading, and answering, they are engaged in multitasking. This probably spreads their attention across multiple tasks, interfering with the creation of a coherent situation representation (Aryadoust et al., 2022). Besides, this might also be related to test-taking strategies when comprehension is obstructed, which was reflected in some verbal reports noting “keyword matching test-taking strategies.” This meant that they often looked for keywords or phrases in the test items, and then matched them with what had been heard in order to locate the answer (Namaziandost et al., 2019). This strategy indicated learners’ use of local-level processing (Field, 2009). In other words, they paid more attention to lexical matches instead of generating a global meaning representation of the texts (Zhai and Aryadoust, 2022).

The second most common barrier is a difficulty in understanding a listening text with many unfamiliar words (B2). Here, unfamiliar words not only involve those that are not acquired in written or oral form, but also those that sound unfamiliar. The current finding is in line with many studies which also proved that vocabulary-related problems prohibited the proper understanding of the listening content (e.g., Goh, 2000; Graham, 2006; Cao et al., 2016; Namaziandost et al., 2019; Alharbi and Al-Ahdal, 2022). In the latest listening barrier study conducted by Alharbi and Al-Ahdal (2022), students confessed to having difficulties in identifying the oral form of familiar words, making it harder to activate the relevant phonological and semantic information. Similarly, Goh (2000), Graham (2006), and Namaziandost et al. (2019) also found that most problems reported by learners were associated with vocabulary knowledge. For instance, students may miss the key information of the listening material when they are preoccupied with recalling spoken words or with new terminology, and this may interfere with the ongoing cognitive process. When examining the verbal report data, the authors found this interference was especially true in long passage listening tasks. Based on this, it is apparent that vocabulary and word recognition play a significant role in listening comprehension (Aryadoust, 2017).

The current results reveal that learners rarely possess barriers in identifying speech, which seems to conflict with Nushi and Orouji (2020) who found that the most significant listening difficulties are pronunciation-based ones such as “connected speech” and “not familiar with phonological features like assimilation or deletion of sounds.” One possible reason may be the different participants and research methods in Nushi and Orouji’s study. Their investigation was based on teachers’ views on EFL learners’ listening difficulties through questionnaire and semi-structured interviews.

The findings of this study suggest that EFL learners need to improve their spoken word recognition and their ability to synthesize a global meaning representation of the texts. Once they are aware of these potential problems, they can take tailored remedial actions to cope with them (Graham, 2006). Furthermore, by targeting the problematic areas that affects their comprehension most, instructors could make use of limited teaching time more profitably (Goh, 2000). Ideally, researchers and teachers should work closely to address these barriers and so help learners enhance their listening comprehension ability.

### 5.2. Relationships between listening barriers

The tetrachoric correlations and item profiles make it possible to uncover the relationships between different barrier attributes. The results of this study confirm that there are both compensatory and non-compensatory relationships between the listening barrier attributes. The exploration of their interactions is also possible because the saturated Bug-GDINA model can capture both compensatory and non-compensatory mechanisms (Chen et al., 2013) and fits the data best. In other words, it contains multiple latent traits in such a way that failure on an item (or a task) requires multiple barriers or their interactions. For example, barriers B2 and B4 could either stand alone or combine together to hamper the success of listening cognitive processing (as in the case of item 3 profile in Table 6). One possible reason may lie in the intermediate listening proficiency of our participants. The previous studies reported that due to the limited linguistic knowledge, less-skilled listeners rely primarily on bottom-up (i.e., phonetic and lexical levels) processing (Rost, 2016; Min and Xiong, 2019), so they are more likely to encounter challenges with lower-level processing (Vandergrift and Baker, 2015). More proficient learners are often able to flexibly shift between top-down and bottom-up processing to activate linguistic and contextual information simultaneously (Nix, 2016; Furuya, 2021). The intermediate learners in the current study would less likely experience the lower-level processing difficulties such as phonetic perception, although the vocabulary recognition barrier is still commonly present. On the other hand, compared with highly proficient counterparts, learners in this study still encounter difficulty in higher-level processing such as understanding semantic meanings (B4). This can also be reflected in the personal attribute pattern of the learner No. 12, who ranks at the intermediate level (63% of the total) and demonstrates listening barriers mainly in vocabulary recognition (B2) and semantic meanings (B4). This result informs teachers that the two barriers should be tackled as a matter of priority, and even concurrently, in their remedial instruction for EFL intermediate learners.

In addition, this study revealed that the barrier in understanding syntactic or semantic structures (B3) alone does not hinder listening success unless combined with other barriers such as B5. This can be explained by the example of item 13 in Table 6, which demonstrates compensation of the two. This finding seems to be consistent with the opinion of Nix (2016), stating that though bottom-up processing plays a fundamental role in listening, the mastery of top-down processing is still indispensable, which indicates the interaction between lower and higher processing in listening (Min and Xiong, 2019). Specifically, comprehending the syntactic and semantic structures often involves a bottom-up process (Field, 2013; Rukthong and Brunfaut, 2019), and if these structures are not perceived, recognized or comprehended, learners prefer to utilize background knowledge and contextual cues such as prosody to compensate for the loss in lower-level processing (He and Xiong, 2021; He et al., 2022). In other words, learners can strategically adopt top-down processing to facilitate inference-making tasks (Min and Xiong, 2019; Chen and Chen, 2021). Another possible reason may be correlated with the difficulty level of listening barrier attributes. According to the diagnostic results of attribute prevalence, the average mastery probability of learners on B3 and B5 is 39.46 and 37.38% respectively, indicating both barriers are relatively less difficult or less likely to be encountered. As Ravand (2016) states, the interrelationship between skill attributes may vary with their difficulty level, and the interaction between skills with lower difficulty tends to be more compensatory. Based on the results of this study, it would also be possible for barrier attributes with less difficulty to demonstrate a compensatory nature.

## 6. Conclusion

This study addresses Chinese college EFL learners’ listening barriers by cognitively diagnosing their incorrect options through the application of Bug-CDMs. It advances the curent CTT- or IRT- based uni-dimensional barrier research to the psychometric multi-dimentional CDA-based research, which can also reflect the attribute barriers’ interactions within an item. Considering Bug-CDMs’ limited application in EFL listening assessment, this study can be seen as a significant step toward their feasibility and usefulness in L2 research.

However, the study is not without limitations. First, we did not investigate barriers across learners with different proficiency levels. For better understanding and interpretation of barriers, future research is recommended to address this area. Second, this study focuses only on the response level of an item, i.e., whether the answer is correct or incorrect, not the information at the individual option level. Considering this, one more step is desired to diagnose students’ weaknesses from the option level, which may help improve the diagnostic accuracy, thereby contributing to the more targeted and in-depth feedback.

Despite the aforementioned limitations, the current study has great implications in that it confirmed that Bug-CDMs can offer valuable insights into how listening barriers are distributed among EFL learners, and how these barriers interact in complex ways. The findings would inspire EFL teachers to provide effective remedial instructions. At the same time they inform the high-quality MCQ test design and development.

## Data availability statement

The raw data supporting the conclusions of this article will be made available by the authors, without undue reservation.

## Ethics statement

Ethical review and approval was not required for the study on human participants in accordance with the local legislation and institutional requirements. The patients/participants provided their written informed consent to participate in this study.

## Author contributions

YM: study design, data collection, supervision, proposal writing, multiple draft writing, and revision. YW: final data analysis, original proposal writing, multiple draft writing, and revision. NZ: data collection and preliminary data analysis. All authors contributed to the article and approved the submitted version.

## Funding

This work was supported by the National Social Science Fund of China (Grant No. 16BYY096), the NEEA and BC English Assessment Research Grants (Grant No. EARG2020007), and Shaanxi Province Social Science Foundation (Grant No. 2022K005).

## Conflict of interest

The authors declare that the research was conducted in the absence of any commercial or financial relationships that could be construed as a potential conflict of interest.

## Publisher’s note

All claims expressed in this article are solely those of the authors and do not necessarily represent those of their affiliated organizations, or those of the publisher, the editors and the reviewers. Any product that may be evaluated in this article, or claim that may be made by its manufacturer, is not guaranteed or endorsed by the publisher.
