# Supplementary material for: Cognitive diagnostic assessment of EFL learners’ listening barriers through incorrect responses
Source: Front Psychol. 2023 Aug 17;14:1126106. doi: 10.3389/fpsyg.2023.1126106 (PMC10469845; doi:10.3389/fpsyg.2023.1126106)
Supplement: Supplementary file 1 [file Data_Sheet_1.PDF]

# Appendix One: Diagnostic Listening Test

**Section I**

**Directions:** *In this section, you will hear several short conversations or statements. At the end of each one, a question will be asked about what was said. You should choose the correct answer from the four choices marked A, B, C, and D. Then mark the corresponding letter on the Answer Sheet.*

1. A) He is drowning. C) He is driving.
   B) He is drawing. D) He is dreaming
2. A) Whether it is made of light straw. C) Whether it has a long string.
   B) Whether it is light and strong. D) Whether it has long stripes.
3. A) The bus was late. C) She got up late.
   B) Her clock was slow. D) She forgot her class.
4. A) She went out jogging. C) She went out for a walk.
   B) She had no more milk. D) She was delivering milk.
5. A) John failed the final exam. C) John has passed the final exam.
   B) John is absent from school. D) John keeps studying though tired.
6. A) $24 C) $64
   B) $30 D) $56
7. A) They missed the train because of the bad traffic.
   B) They arrived at the railway station just in time.
   C) They barely caught the bus to the railway station.
   D) They had a traffic accident on the way to the station.
8. A) She could survive the earthquake without help.
   B) She couldn't live by herself after the earthquake.
   C) She survived the earthquake and saved the man too.
   D) She would have died if the man hadn't arrived in time.
9. A) The woman didn't know the new teacher.
   B) The new teacher has been sick for two days.
   C) The man didn't understand the woman's question.
   D) The man hasn't met the new teacher yet.
10. A) Easter. C) Valentine's Day.
    B) Halloween. D) Independence Day.
11. A) He'll go to the party with the woman. C) He has changed his plans.
    B) He won't meet the man at the party. D) He has to work late.

**Section II**

**Directions:** *In this section, you will hear a long conversation. At the end of the conversation, you will hear some questions. Both the conversation and the questions will be spoken only once. After you hear a question, you must choose the best answer from the four choices marked A, B, C and D. Then mark the corresponding letter on the Answer Sheet.*

**The following questions are based on the conversation you have just heard.**

12. A) They look forward to the class trip. C) The school sports event is on Friday.
    B) The weather is perfect for a picnic. D) The professor will cancel his class.

13. A) She enjoys it.
    B) She thinks it's too tiring
    C) She prefers outdoor activities.
    D) She's worried it will be too difficult.

**Section III**

**Directions:** *In this section, you will hear several short passages. At the end of each passage, you will hear some questions. Both the passage and the questions will be spoken only once. After you hear a question, you must choose the best answer from the four choices marked A, B, C and D. Then mark the corresponding letter on the Answer Sheet.*

**Passage One**

**The following questions are based on the passage you have just heard.**

14. A) The three key reasons why people should volunteer.
    B) The three main areas people volunteer in.
    C) The influence of volunteer activities.
    D) The major ways of volunteering.
15. A) It is needed when wars break out.
    B) It is busy in dealing with urgent problems.
    C) It is under a heavy burden to handle local issues.
    D) It is a little slow in dealing with local problems.
16. A) Helping others brings positive emotions.
    B) Volunteering benefits the receivers more.
    C) Helping others means power.
    D) Everyone needs help and friends.

**Passage Two**

**The following questions are based on the passage you have just heard.**

17. A) They are very generous in giving gifts.
    B) They refuse gifts when doing business.
    C) They regard gifts as a token of friendship.
    D) They give gifts only on special occasions.
18. A) They enjoy giving gifts to other people.
    B) They spend a lot of time choosing gifts.
    C) They have to follow many specific rules.
    D) They pay attention to the quality of gifts.
19. A) Gift-giving plays an important role in human relationships.
    B) We must be aware of cultural differences in communication.
    C) We must learn how to give gifts before going abroad.
    D) Reading extensively can make one a better gift-giver.
